# Injectable GSK3 inhibitor (Tideglusib) hydrogel versus enhanced β-tricalcium phosphate in tibial bone healing (in vitro and in vivo study)

**DOI:** 10.1007/s10266-025-01203-4

**Published:** 2025-09-21

**Authors:** Mahitabe Elgamily, Mona Denewar, Ahmed A. Emam, Basma Hamed Othman, Nessma Sultan

**Affiliations:** 1 Faculty of Dentistry, Mansoura University, Mansoura, Egypt; 2 Faculty of Dentistry, Mansoura National University, Gamasa, Egypt; 3 Medical Experimental Research Center (MERC), Faculty of Medicine, Mansoura University, Mansoura, Egypt

**Keywords:** Tideglusib hydrogel, Beta tricalcium phosphate, Bone regeneration

## Abstract

This study was carried out to assess the potential effect of a glycogen synthase kinase-3 inhibitor tideglusib hydrogel formulation on bone regeneration and to compare it with Ethoss a beta tricalcium phosphate alloplastic bone substitute mixed with calcium sulphate in vitro and in vivo. Bone marrow mesenchymal stem cells (BMMSCs) were isolated from New Zealand white rabbit tibias and characterized by flowcytometric analysis. Sodium alginate hydrogel loaded with tideglusib was formulated and underwent characterization by scanning electron microscopy, the fourier transform infrared and zeta potential. Extraction media from both tideglusib hydrogel and Ethoss were prepared then MTT, alizarin red (ARS) and scratch wound assays were performed. Two bone defects were trephined in tibias of eighteen adult healthy rabbits, which were classified into three groups: group 1, bone defects were left untreated; group 2, each defect was filled with tideglusib hydrogel; group 3, each defect was filled with Ethoss. Animals were sacrificed after three and six weeks. Histological and immunohistochemical analyses using alkaline phosphatase (ALP) and osteopontin (OPN) were performed to assess bone regeneration. The tideglusib hydrogel group expressed higher mineral deposition than Ethoss group using ARS but without significant difference, stronger stimulatory effect on the migration of BMMSCs was also evident with significant narrowing of the wound area. New bone tissue was progressively seen bridging and filling the defects with significantly higher ALP and OPN expression in hydrogel group. Tideglusib hydrogel formulation exhibited superior bone regenerative potential in comparison to beta tricalcium phosphate alloplastic bone substitute.

**Graphical Abstract:**

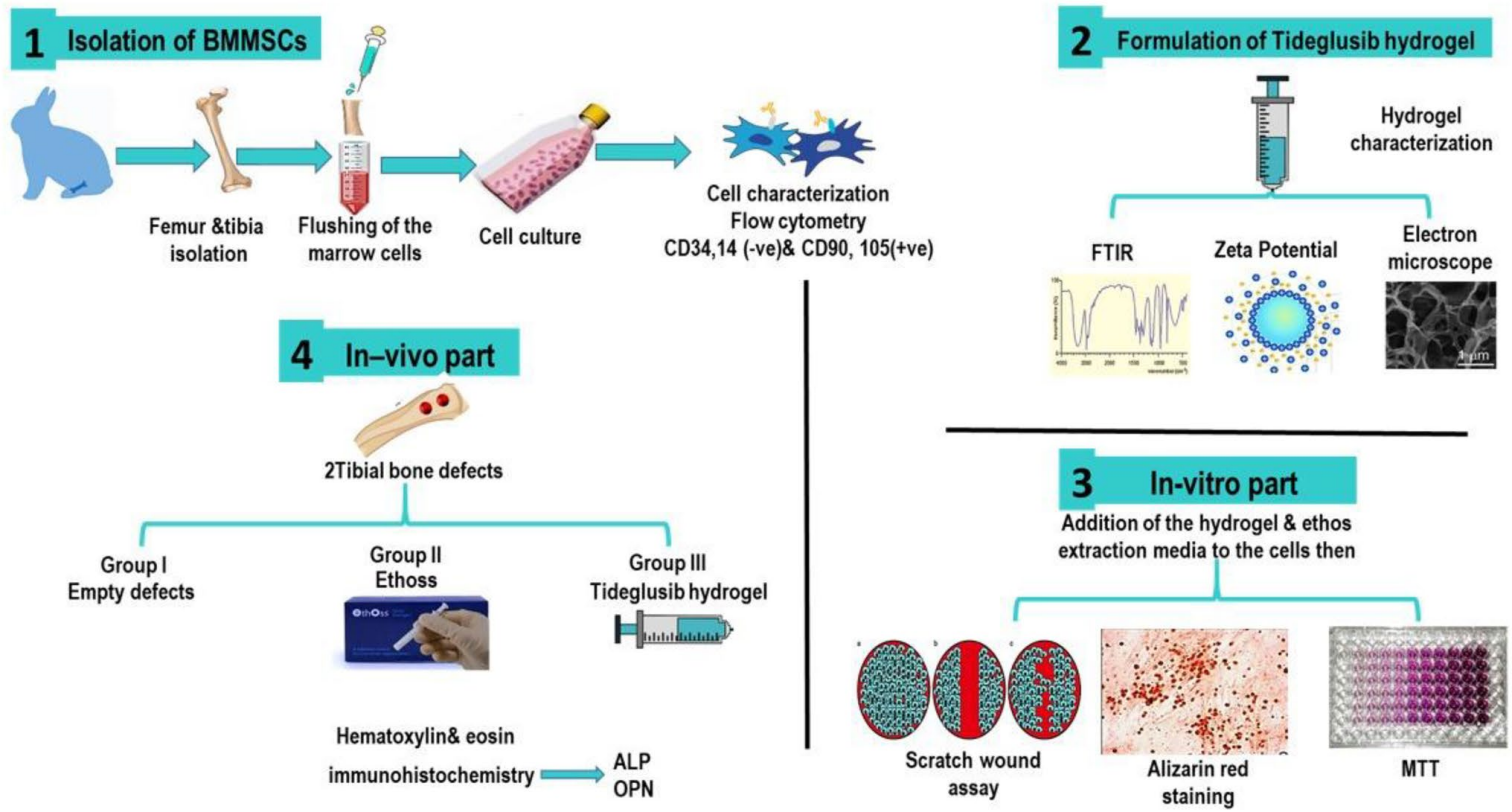

## Introduction

Bone is a large dynamic tissue in the human body with numerous crucial physiological functions [1]. Various traumatic events may lead to cranio-orofacial bone defects such as trauma, tooth loss, sinus pneumatization, periodontal diseases, cysts, tumors and infection. Although bone tissue has a self-regenerative property, regeneration cannot take place when defects exceed a certain size [2], so defects should undergo reconstruction to fill empty spaces and preserve the normal anatomical frame and also to render aesthetic restorations and dental implants possible [3].

Bone grafting is a challenging process because it affects patient's quality of life. Various grafting materials have been utilized including allografts, autografts, xenografts and synthetic grafts. The autogenous grafts are considered as the gold standard for bone regeneration as they have superior osteogenic, osteoinductive and osteoconductive properties [4, 5]. Despite of all these valuable properties of autogenous grafts, pain, bone resorption and morbidity in the donor site are great limitations [6]. None of the available products offer all of the ideal criteria as a bone substituting material including lowest mortality, angiogenic potential, easy handling, low immunogenicity and inexpensive cost. Therefore, there is a great need for the formulation of new grafts that substitute bone tissue [7].

Activation of various signaling pathways is required for the development and the repair responses taken by the organisms. Wnt /b-catenin pathway is an important signaling pathways, which is activated early after tissue damage and is a mandatory step for inducing the repair [8]. Several studies reviewed that Wnt signaling is important for osteoblast differentiation with an enhanced effect in bone healing [9–11]. Other studies proved that Wnt signaling suppression has an association with bone loss and decreased bone regeneration [12–14]. Wnt protein firstly binds to its cellular receptors with subsequent transcription of target genes (e.g. Axin) of Wnt signaling. Stimulation of Wnt signaling upregulates osteoblastogenesis [15].

Glycogen synthase Kinase-3 (GSK-3) molecule is a milestone in regulating cellular division cycle. Its activation leads to suppression of Wnt/b-catenin pathway thus inhibiting osteoblastogenesis [16, 17]. So, the development of novel GSK-3 inhibitors is of high importance [18–21]. Tideglusib is a selective and irreversible non-ATP competitive GSK-3 inhibitor, it is utilized for treating some neurologic diseases e.g. Alzheimer disease [22, 23]. Recently, tideglusib was used to effectively stimulate reparative dentin formation [8] increasing the expression of Wnt signaling to 3 folds higher and the newly formed dentin tissue expressed a comparable radiopacity to that of surrounding primary/secondary dentin [24, 25]. Dentin and bone are both hard tissues that share many similarities, so researchers started to assess the role of tideglusib in bone damage as well. Most of the previous studies used the tideglusib dissolved in DMSO and soaked into collagen or gelatin sponge as a drug vehicle [26, 27].

Hydrogel is a type of injectable scaffolds that represents a new era in regeneration as they can fill the non-uniform defects while homogenously deliver the bioactive molecules in a minimally invasive approach. Hydrogel also express special viscoelastic properties similar to the connective tissue of the extracellular matrix with high water affinity enabling them to absorb large amount of biological fluids with preservation of its structural integrity in aqueous media accompanied by gradual degradation for controlled drug release, so we decided to study the effect of tideglusib in a new hydrogel formulation different from the previously studied formulations of the drug [28].

Beta-tricalcium phosphate (β-TCP) is an extensively utilized alloplastic bone substitute material which is biocompatible and osteoconductive as it is similar to cancellous bone in compressive strength and resorption rate. By mixing calcium sulphate (CS), which has excellent properties such as bonding between host bone and bone graft, with β-TCP an alloplastic compound named Ethoss was developed. This compound hardens in place and adheres to the host bone and helps restore the shape of the augmented site by serving as a stable scaffold [29, 30].

So, our study was conducted to assess the osteoinductive ability of tideglusib new formulation (loaded in sodium alginate hydrogel) and to compare it with Ethoss (alloplastic bone substitute). The study explores the cytotoxicity and mineralized tissue formation capacity of the formulated hydrogel in comparison to Ethoss on BMMSCs in vitro. The in vivo part investigates the regenerative potential focusing on the capability of the formulated hydrogel to promote osteogenesis in rabbit tibial bone defects.

## Material and methods

### Isolation and characterization of BMMSCs

BMMSCs were isolated from two New Zealand white rabbit’s femurs. All connective tissues were cleaned from sectioned bones which were collected in 10 ml transport medium containing basic medium Dulbecco modified essential medium/F12 (DMEM/F12) with L-glutamine (Life science, UK) supplemented with 20% fetal bovine serum (FBS), in addition to penicillin and streptomycin (100 μg/mL each) (Biowest, united states). In a biohazard laminar flow hood, each bone was flushed with 10 ml of complete culture medium to collect BMMSCs. The complete culture medium contained DMEM/F12 with 20% FBS, 1% antibiotic–antimycotic. Filtration was then performed using 70 *μ*m nylon cell strainer into T-75 tissue culture flask (Greiner Bio-One International, Austria). Incubation was done at 37 °C (5% CO2). This was followed by removal of non-adherent cells by changing the medium after 24 h. Changing of culture medium was done every 48–72 h.

To study surface molecular expression in BMMSCs, 5 × 10^5^ cells were fixed (for 15 min) with 4% paraformaldehyde and underwent washing in phosphate buffer saline (PBS) and labelled with primary antibodies CD105, CD 90, CD 34 and CD14 for 60 min. Then, cells were labelled with fluorescein isothiocyanate-conjugated secondary antibodies for 45 min. The percents of cells that stained positive for CD 90, CD105 and that stained negative for CD 34, CD14 were assessed using FACS calibre flow cytometry (BD Immunocytometry Systems).

### Formulation of Tideglusib hydrogel

Sodium alginate was prepared in accordance with the previously published articles [31, 32]. To produce 5% (w/v) of sodium alginate, 0.125 g of sodium alginate (Sigma-Aldrich, St. Louis, MO, US) was dispersed in 2.5ml distilled water and glycerol. This solution underwent filtration using 0.45-μm sterile syringe filter. After that, tideglusib (Sigma Aldrich SML0339, CAS.No 865854–05-3, United Stated) was dispersed in sodium alginate solution at a ratio 1:1. The hydrogel was formed through dripping the solution into CaCl2 5% (w/v) solution (Sigma-Aldrich, St. Louis, MO, US). After hydrogel formation, it was transferred into a syringe to obtain an injectable hydrogel mass.

### Characterization of the formulated hydrogel

#### SEM

SEM (Jeol JSM 6510, Jeol, Peabody, MA) was utilized to assess individual hydrogels' cross-sectional morphology. Following gold-sputtering, hydrogel was placed on SEM stubs and analysed at 500 × magnification with an accelerating voltage of 15 kV.

#### Size and zeta potential-surface charge

Before measurement, 1 mg of the gel underwent dispersion in deionized water (10 ml) and mixed well. Particles were characterized for size and size distribution, as regards the average volume diameters and polydispersity index (PDI), by photon correlation spectroscopy utilizing particle size analyzer Dynamic Light Scattering (DLS) (Zetasizer Nano ZN, Malvern Panalytical Ltd, UK) at fixed angle of 173° at 25° C. Analysis was performed in triplicate. Zeta potential was determined using the same equipment.

#### The FTIR

The FTIR spectra were recorded in the wavenumber range from 4000 to 400 cm − 1 at a resolution of 2 cm^−1^ at 25 °C, on a FT-IR spectrophotometer (iS10 Nicolet Model, Thermo Fisher Scientific, USA).

### Evaluation the cytotoxicity of tested materials

#### Extraction medium preparation of the tested materials

Ethoss [a self-hardening material, containing β-TCP and CS (65% and 35%, respectively) in a powder form] was purchased from Ethoss Regeneration Ltd., Silsden, United Kingdom. The material was prepared following the manufacturers’ instructions and all mixtures were prepared in sterile conditions and incubated in 24-well plates for 24 h till complete setting. In sterile condition, 1 ml culture medium was added to either Ethoss or tideglusib hydrogel and underwent incubation for 24 h at 37°C in a 5% CO2 atmosphere. The extraction medium from each material was collected and underwent filtration (0.22 µm filter unit) and was kept in − 20 °C till being utilized.

#### MTT assay

The cytotoxic effect of the tested materials was conducted utilizing MTT assay (MTT Cell Growth Kit; Chemicon, Rosemont, IL) after 72 h of culture. Briefly, BMMSCs were cultured in culture media DMEM + 20% FBS. 24 h after seeding, the culture medium was removed and replaced by different concentrations of the extraction medium of the tested materials (100%,80%,60%, 40%, 20%). A dose–response relationship was examined through diluting the extraction medium with complete culture medium to obtain the different concentrations. 72 h after incubation, MTT was added at 1 mg/mL and cells underwent incubation for 4 h. After that, culture medium that contained MTT was removed with and 100 μL of dimethyl sulfoxide was added to release formazan. The absorbance at 570 nm was measured utilizing microplate reader (ELx800; Bio-Tek Instruments, Winooski, VT) and Abs 690 as a reference wavelength. Each condition was analysed in triplicate.

### Scratch assay

BMMSCs (2 × 10^5^ cells/well) were seeded into 6-well plates and cultured in complete culture medium for 24 h. A sterile pipette tip was used to create a scratch, and the extraction medium replaced the culture medium and cultured for 24, 48 h. Cells seeded in osteogenic medium, Sigma Aldrich (ascorbic acid 50 μg/ml, β-glycerolphosphate 10mM and Dexamethasone 0.1 μM) were used as positive controls in this assay. The “wound” was observed using a phase contrast microscopy at 0, 24, and 48 h. An image was utilized for determination of the healing area (scratch width) at same locations [33].

### Alizarin red staining (ARS)

BMMSCs were seeded into 6-well plates at concentration 5 × 10^4^ cells/well. After 24 h, the material’s extracted medium replaced the media and cultured for 2 weeks. ARS was done at day 14. Cells underwent fixation with methyl alcohol and washing was done using deionized water. AR solution (1% w/v) was incubated with samples for 20 min, then the wells underwent washing with deionized water three times. Cells were visualized under phase contrast microscope.

### Animal selection

Animal experiments were carried out at the Medical Experimental Research Center, Mansoura Faculty of Medicine. The protocol was accepted by Mansoura University’s ethical committee; Faculty of Dentistry, Egypt (code No. MU-ACUC (DENT.R.24.09.13). Appropriate housing of the animals was done in separate cages with free access to food and water.

#### Sample size calculation

Total of twenty rabbits were used in this experiment; two rabbits were used for BMMSCs isolation, and eighteen adult healthy male New Zealand white rabbits were utilized after exclusion of female rabbits or previously-treated, unhealthy, and low-weight rabbits. One way ANOVA was utilized to analyze variance; according to Cohen, eighteen samples was sufficient to detect the effect size of 0.89 at a power (1-β = 0.90) of 80% at a significance probability level of P < 0.05. The sample size of 36 statistical units was utilized, which each group included 6 samples. Sample size underwent calculation using G*Power V 3.1.9.6 (Cohen),13 where f is the effect size = 0.89; α = 0.05; β = 0.1; Power = 1-β = 0.90) [34]. Based on previous experience, 12 defects (6 animals) in each group were sufficient [35, 36].

#### Experimental design and grouping (Fig. 1)

**Fig. 1:**
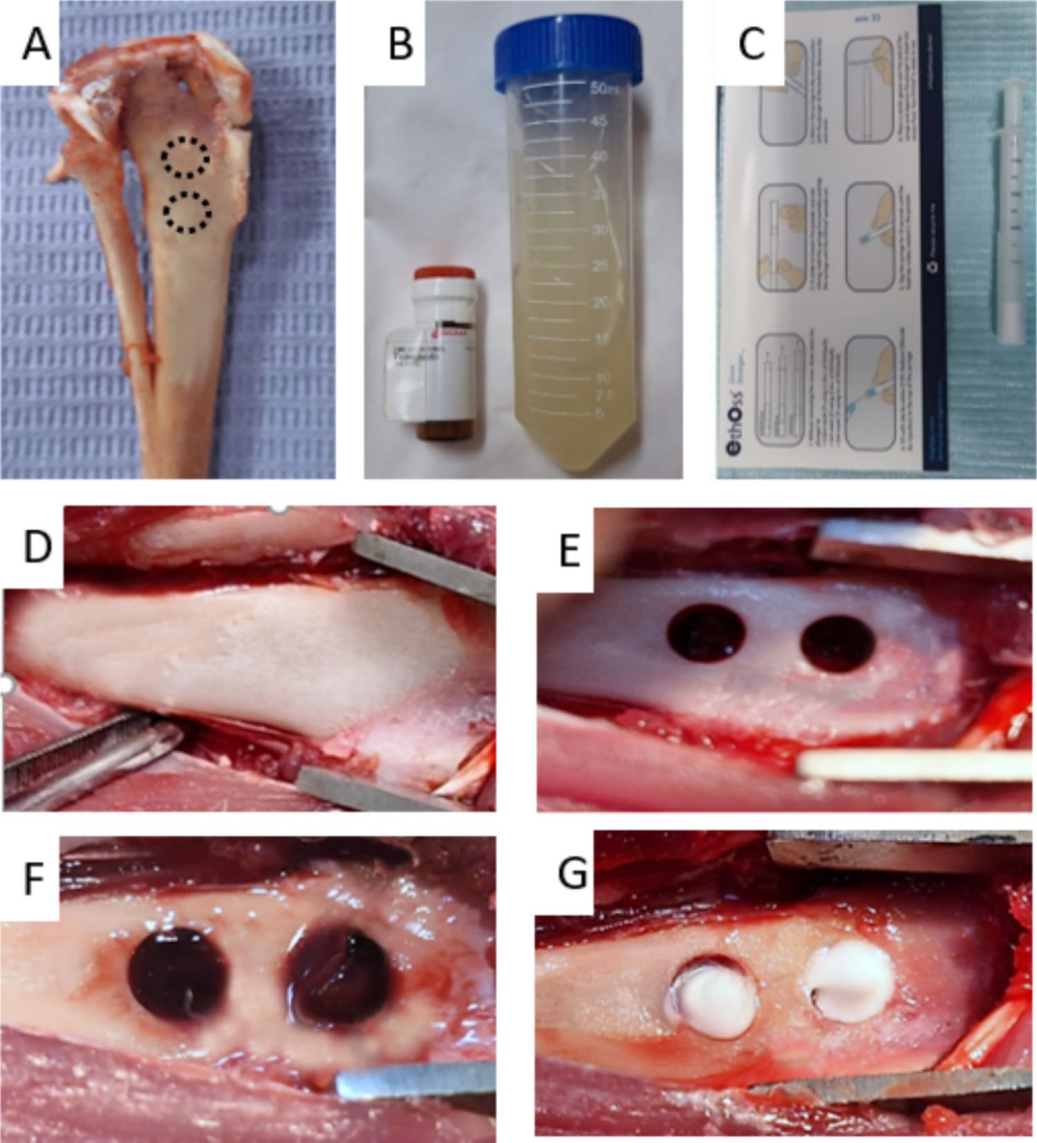
Photographs showing A Anatomical Position of the two surgical osteotomies in the proximal tibial metaphysis, B Tideglusib hydrogel, C Ethoss; composed of β-TCP and CS (65% and 35%, respectively), loaded as a powder form into a sterile plastic syringe, D Surgical site, E The two empty monocortical bony defects 4 mm in diameter in tibial bone, F Defects filled with hydrogel, G Defects filled with Ethoss

Two monocortical bone defects were surgically created in the right tibias of all animals using trephine bur (3i, Palm Beach Gardens, FL, US) 4 mm in diameter obtaining full depth using a low-speed contra-angle hand piece (NSK, Japan). The osteotomies were performed in the tibial metaphysis, specifically in the proximal third of the tibia (proximal metaphysis of the tibia) at 250 rpm speed, with saline cooling. Irrigation of cavities was carried out utilizing a plastic syringe containing normal saline. Drying was then done with sterile cotton pellets. Tideglusib hydrogel was then injected into the cavity, 50 μl was used to completely fill the circular defect, while Ethoss was inserted and compressed in the cavity using a bone plunger.

The two defects per rabbit were filled with the same tested material. This experiment included 3 groups (each is 6 animals) based on the treatment; Group I (controls), manual pressure haemostasis was applied with a gauze and there was no treatment for the bone defect. Group II addressed bone defect with tideglusib hydrogel as a grafting material. Group III Ethoss was applied to fill bone defect.

Specimens were dissected 3 and 6 weeks post-surgery and were fixed in 10% formalin. In each group, six animals were utilized; three rabbits were scarified 21 days post-surgery, while the other three animals were scarified 6 weeks post-surgery. 10% ethylenediaminetetraacetic acid (EDTA) was used to achieve decalcification. Slides were numbered to be blindly evaluated without knowing the material utilized.

#### Surgical procedures

Ketamine hydrochloride and xylazine (20 and 25 mg/kg respectively) (ADWIA Co. SAE 10th of Ramadan city Egypt) were intraperitoneally injected to achieve general anesthesia. Anesthesia (2% mepivacaine HCl) was also injected locally into the surgical site for improvement of local homeostasis and post-operative analgesia [37, 38]. Disinfection was accomplished with betadine and alcohol after shaving of the tibial skin followed by a sharp 6 mm longitudinal incision along the proximal right tibia. Mucoperiosteal elevator was carefully used for detachment of the periosteum and bone exposure[39].

#### Postoperative medication

The overlying soft tissues and skin were stitched in layers utilizing resorbable 3–0 Vicryl sutures (Ethicon®, Johnson & Johnson, Somerville, NJ, US). Each incision site was cleaned with betadine and sterilized gauze. Antibiotic drugs (cefotaxime 1g vials, Egyptian International Pharmaceutical Industries, Ramadan, Egypt) were administered once daily. Analgesic drugs (Voltaren 75 mg/3 mL Novartis, Giza, Egypt) were given for 48h post-operatively. Animal sacrifice was done at 3 and 6 weeks post-surgery. After that, tibial bones containing healing areas were harvested and underwent fixation in formalin (10%) for 24 h [40].

### Histologic examination

A cutting machine was utilized to harvest the area of interest. Tissues were decalcified through immersion in a EDTA solution (pH 7.2): tibial samples usually require 3–4 months for appropriate tissue processing. After that, samples were embedded in paraffin, stained with haematoxylin and eosin (H&E) and histological analysis was done by microscopy (Olympus Co., Tokyo, Japan).

#### Immunohistochemistry staining

Using the streptavidin–biotin immunoperoxidase approach, anti- Osteopontin OPN primary antibody (1B20, NB110-89062, at a 1:150 dilution Novus Biologicals) and Alkaline phosphatase ALP antibodies (cat# ab224335 at a 1:300 dilution, Abcam) were utilized for the immunohistochemistry study. Histologic slices of 3 µm in thickness were deparaffinized and underwent hydration. To remove formalin, they were soaked for 10 min in a solution of 10% ammonium hydroxide and 95% ethyl alcohol, followed by washing in distilled water. They were then treated with 1% pepsin solution (pH = 1.8) at 37 °C for 1 h for antigen extraction. Subsequently, cleaning of samples was done and they were blocked with 3% H_2_O_2_. Specimens were then washed under running water and distilled water to remove the solution. Incubation was then performed at pH 7.4 in PBS solution for 3 min in dark, with 300 mg of diaminobenzidine as chromogen (3,3-diaminobenzidina; Sigma Chemical CO., St. Louis, MO, US) in 100 mL of PBS solution, pH 7.4. After three minutes of haematoxylin counterstaining, slides underwent dehydration by an increasing ethanol series and were cleaned with xylene, and placed on Permount® (Fischer Scientific, Fair Lawn, NJ, US) to be examined using light microscopy.

### Image analysis

Image analyses were conducted for ARS photomicrographs by calculating the % area of red color expression. Immunohistochemical stained slides were also utilized for image analysis. Three tissue slides were prepared from control and experimental animals at each time point. We captured (at magnification 100 ×) 4 random areas in the middle of defect. So, 12 images from each group per time point were analysed, with calculation of the ratio of new bone formation to the total defect area. Image analysis was done by Intel® Core I7® based computer and VideoTest Morphology®,Russia), which features a specific built-in routine for area and percentage area measurements. Images used for analysis were stained with OPN and ALP. The positive reaction appeared brownish in colour.

### Statistical analysis

Data were analyzed by SPSS software V 26 (SPSS Inc., PASW statistics for windows. Chicago: SPSS Inc.). Quantitative data were represented as means ± SDs for normally distributed data after testing normality by Shapiro Wilk test. Statistical significance was judged at (0.05) level. One Way ANOVA test was used for comparison > 2 independent groups with Post Hoc Tukey test to detect pair-wise comparison.

## Results

### Characterization of BMMSCs

**Fig. 2:**
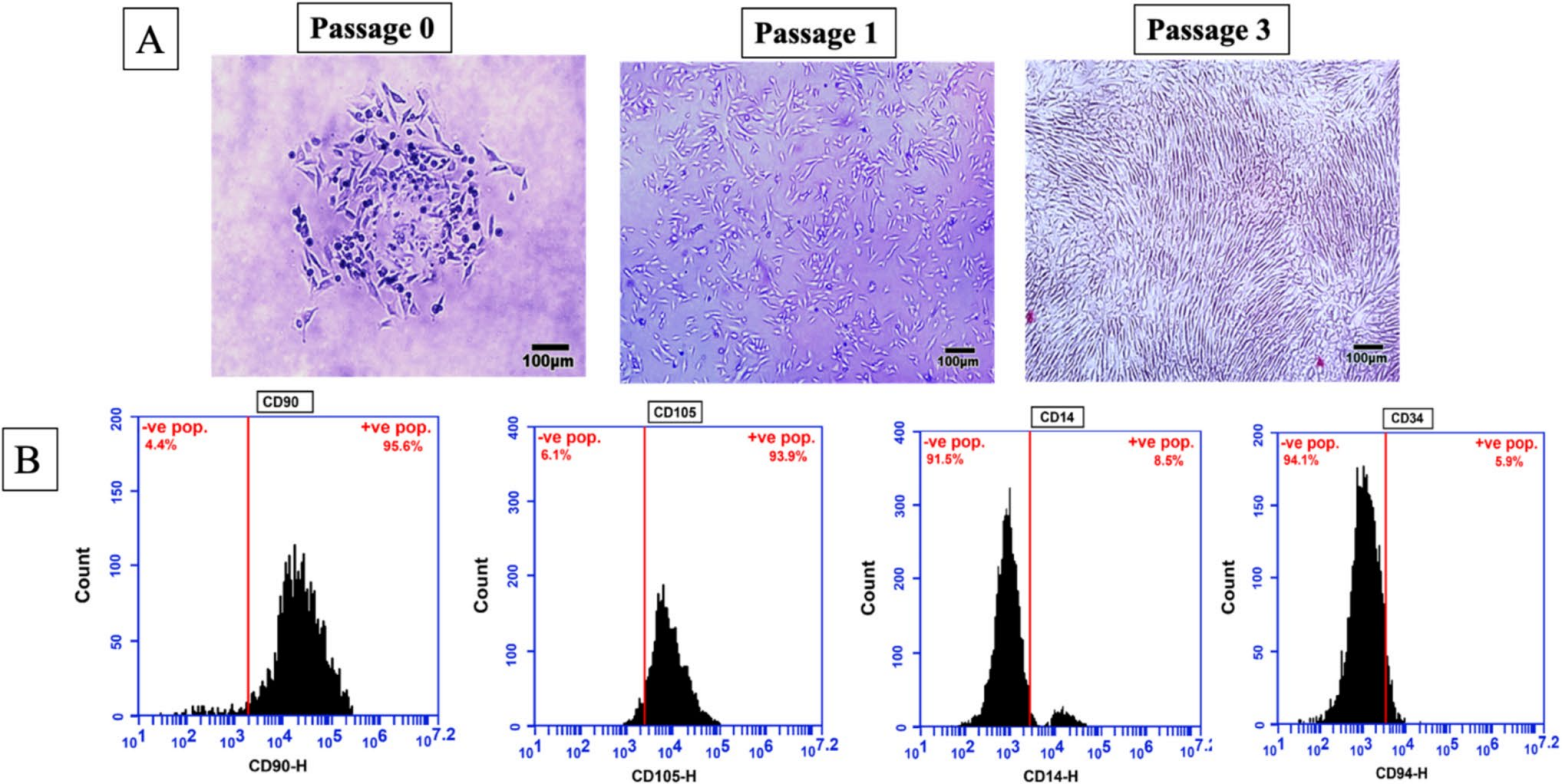
**A** Inverted microscopic photographs showing rabbit’s BMMSCs after isolation where; at P0 shows adherent cells are mostly rounded with few spindle shaped cells. P1 most of the adherent cells showed spindle fibroblast like appearance while after 7 days at P3; growth of the colonies and monolayer cell sheet formation. (scale bar 100 µm). **B** Flow cytometry charts showing phenotype analysis of BMMSCs. Each histogram shows fluorescence intensity as a measurement parameter (on x-axis) and cell count (on y-axis) revealing + ve reactivity to CD 90 and CD 105, and-ve reactivity for CD14 and CD 34

The isolated BMMSCs were evaluated under the inverted microscope. MSCs were distinguished their tendency to plastic adherence. Following subculture, the cells became more homogenous with a spindle-like morphology and relatively thin processes extending from the cell bodies followed by colony formation. At the 3rd passage, the colonies enlarge and fuse forming monolayer and showing confluent appearance. Flow cytometry showed that cells were positive for CD90 (95.6%) and CD105 (93.9%), however they were negative for CD14 (8.5%) and CD34 (5.9%). This confirmed the stemness of BMMSCs (Fig. 2).

### 3.2 Characterization of Tideglusib hydrogel

#### Morphological characteristics

**Fig. 3:**
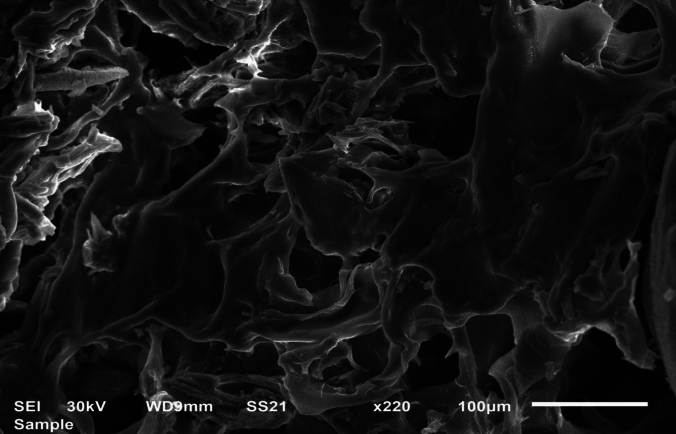
SEM of tideglusib hydrogel showing porous microstructure with interconnected pores (magnification × 220, Bar 100 μm)

SEM micrographs were used to observe the structure of tideglusib hydrogel. Results demonstrated that tideglusib hydrogel have a porous microstructure with interconnected pores (Fig. 3).

#### Size and zeta potential

The formulated hydrogel had a negative surface charge and a maximum peak at − 28.2 ± 1.62 mV. The average size was 227.3 ± 1.2 µm and the PDI was 0.3 ± 0.04.

#### FTIR

The FTIR spectrum confirms the successful incorporation of both Tideglusib and sodium alginate in the hydrogel formulation, as evidenced by the characteristic peaks of both components. It revealed important absorption bands as regards hydroxyl and carboxylic groups. Stretching vibrations of O–H bonds of alginate ranged from 3000–3600 cm − 1. Strong absorption peak around 1600 cm⁻^1^ was also evident this indicates C = O stretching of sodium alginate [41, 42]. Peaks in the 1400–1300 cm⁻^1^ region corresponded to –COO − symmetrical stretching vibration [41].

**Fig. 4:**
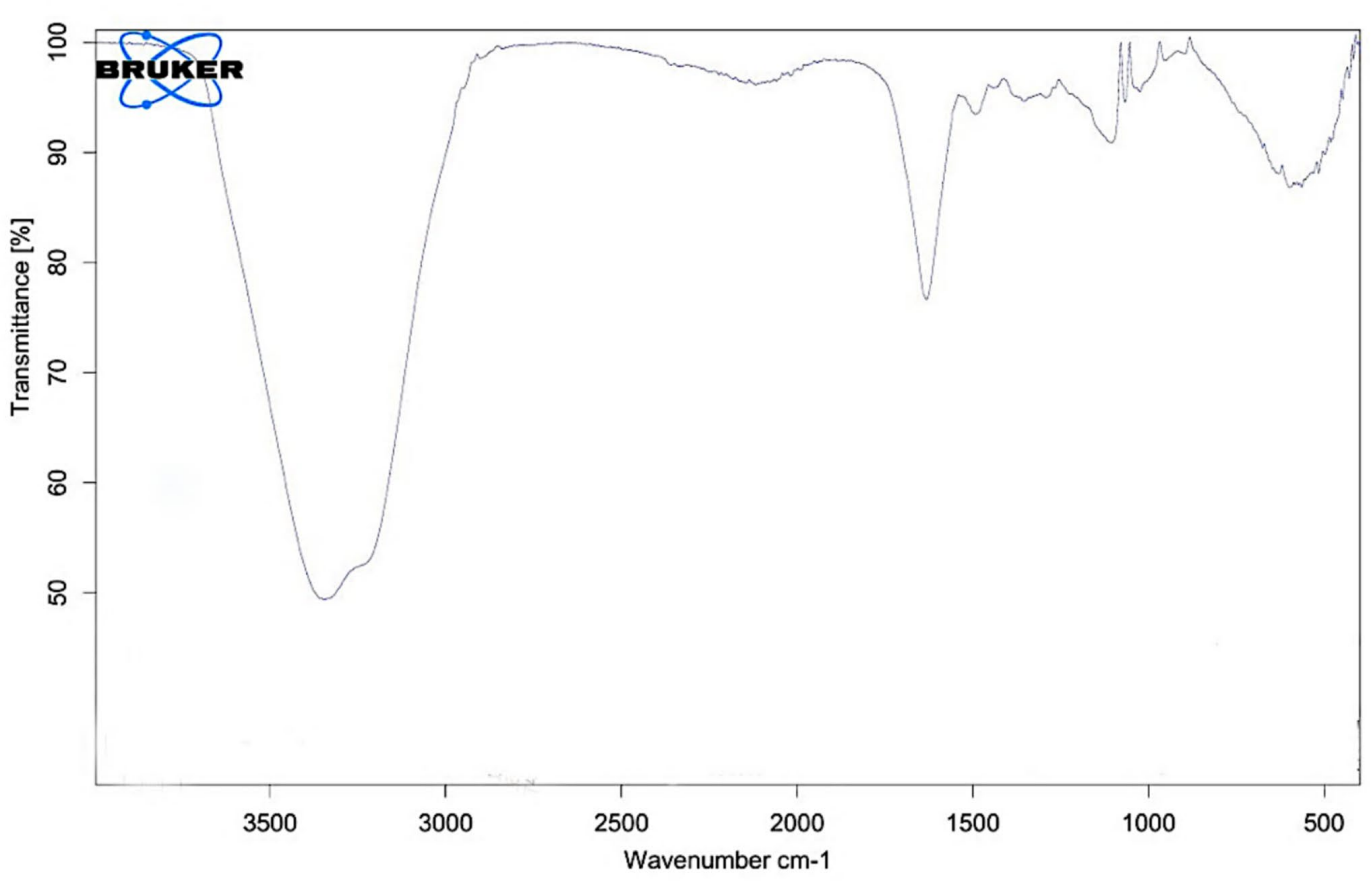
FT-IR spectrum revealing functional groups for sodium alginate hydrogel loaded with Tideglusib

The region between 1200–1000 cm⁻^1^ Showed C–O–C and C-O stretching vibrations typical for the polysaccharide structure of alginate [43] in addition, FT-IR analysis of Tideglusib demonstrated peaks between 1700 and 1500 cm − 1 indicating the existence of Tideglusib with respect to carbonyl-C = O and amide groups [44] (Fig. 4).

### Cytotoxicity assay (MTT)

**Fig. 5:**
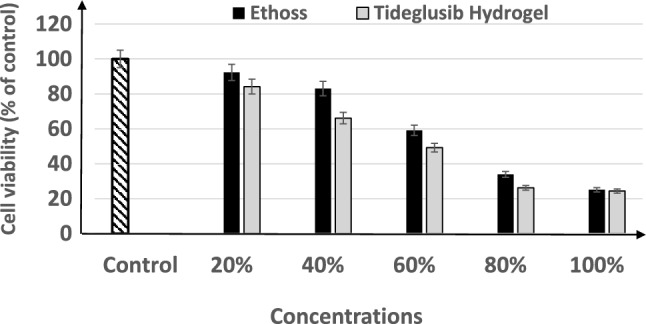
MTT analysis of viable BMMSCs percentage cultured with either tideglusib hydrogel or Ethoss. BMMSCs were exposed to complete medium (control), 20%, 40%, 60%, 80% and 100% hydrogel or Ethoss for 72 h. Data are expressed as mean ± SD; n = 3

MTT assay tested the viability of BMMSCs seeded with different concentrations of tideglusib hydrogel and Ethoss for 3 days (Fig. 5). BMMSCs’ viability in 40, 60, 80% and 100% concentrations of tideglusib hydrogel and Ethoss were significantly lower than control animals (*P* < 0.002, *P* < 0.001, *P* < 0.001, *P* < 0.001 respectively) suggesting their cytotoxicity. However, at 20% concentration, viability did not significantly differ for tideglusib hydrogel and Ethoss (P > 0.099) compared to control animals.

### Scratch wound assay

**Fig. 6:**
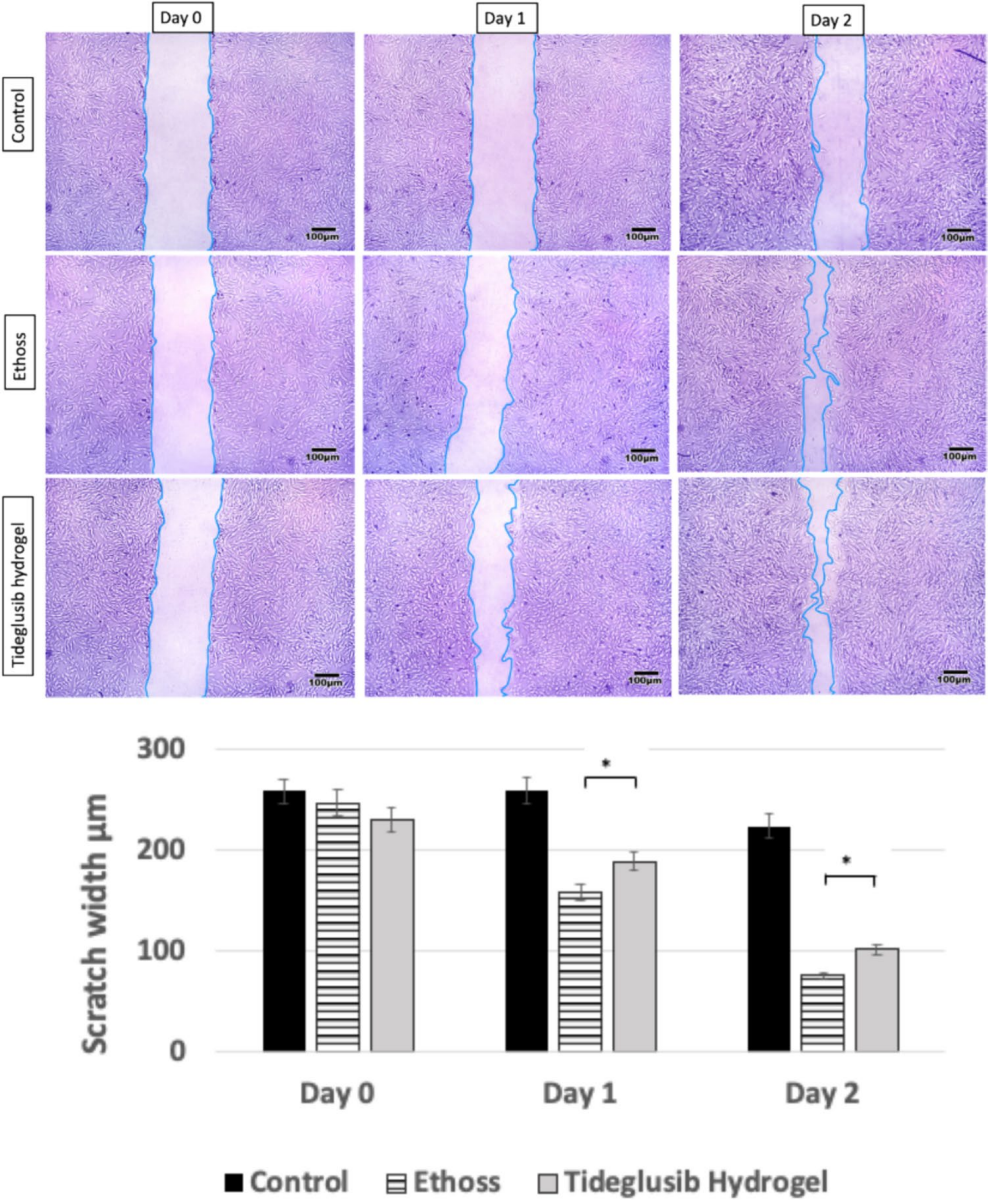
BMMSCs scratch-wound healing assay results evaluating cell migration at 0, 24 and 48 h respectively after exposure to Tideglusib hydrogel and Ethoss extraction medium. (Scale bar, 100 µm). The blue lines indicate the scratch width. The bar chart showing scratch wound healing mean values ± SD among the studied group

The results revealed that tideglusib extraction medium showed a more significant stimulatory effect on BMMSCs’ migration compared to either Ethoss extraction medium or the control medium which was evident by the significant narrowing of the wound area after 24 & 48h (*P* < 0.05) (Fig. 6).

### Alizarin red staining

**Fig. 7:**
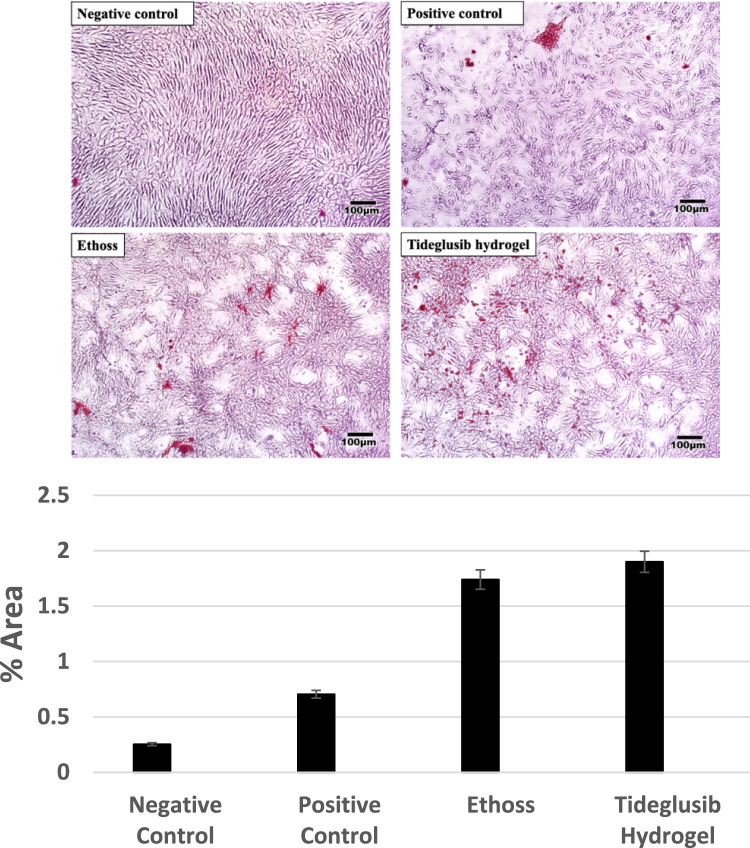
BMMSCs ARS results showing the formation of mineralized nodules after 14 days of culturing with the extraction medium of the tested materials. Bar chart showing ARS mean values ± SD among the studied group

Figure 7 showed the ARS staining photomicrographs at 14 days. Calcium deposits appeared as aggregation of red stain. The Tideglusib hydrogel and Ethoss enhanced the mineralization and calcium deposition significantly higher than either control negative and the control positive group (using osteogenic media). Noteworthy that tideglusib hydrogel expressed higher mineral deposition than what was seen in Ethoss group but without showing statistically significant difference.

### Histologic results

There were no reported mortalities or complications in rabbits. Three- and six-weeks post-surgery, no necrosis, haematomas, or infections were reported. An identification of the defect edges was made possible by the clear contrast between the newly formed bone, which was characterised by numerous osteocytes, and the previous lamellar and less cellular old bone.

#### Haematoxilyin and eosin stain results

##### Three weeks post-surgery

**Fig. 8:**
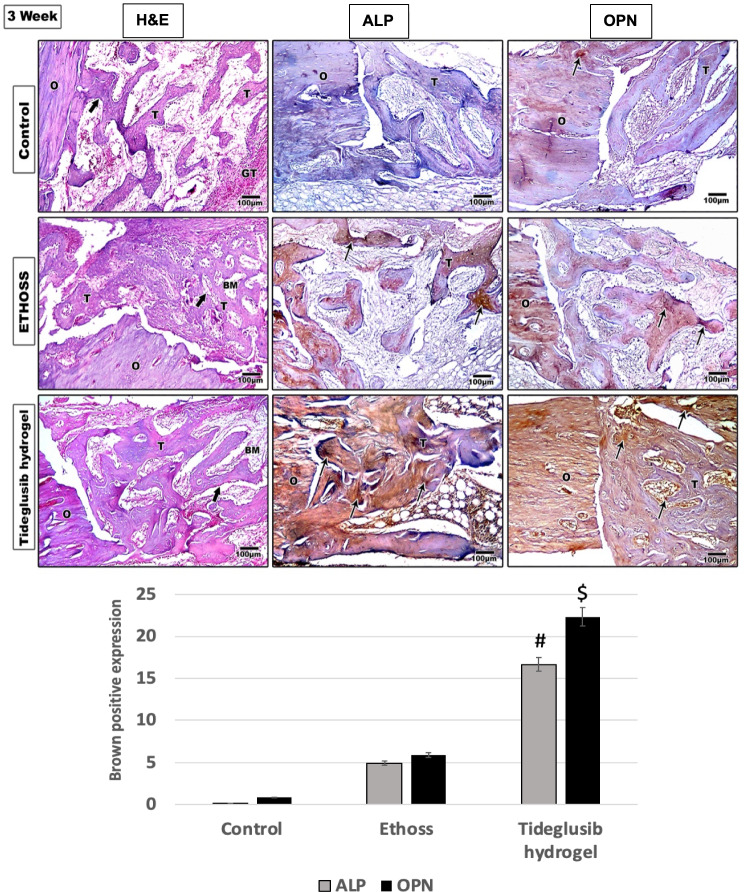
Histologic images of (H&E), Alkaline phosphatase and osteopontin immune staining are demonstrated at 3 weeks after treatment. Granulation tissues with very thin bone trabeculae were observed to be mostly filled in defect areas in control animals. Ethoss group showed bone trabeculae filling the bone defect. The defect filled with tideglusib hydrogel shows the new lamellar bone formation with extensive brown immune expression in ALP and OPN. T: bone trabeculae; O: old bone; BM: bone marrow; GT: granulation tissue. Arrows represent osteoblasts lining in H & E images and ALP and OPN-positive reactions in immune images. Scale bar: 100 μm. The bar chart shows the histomorphometric analysis of ALP and OPN positive expressions at 3 weeks Data are represented as means ± SEM where # and $ means significant in relation to Ethoss and control groups *P* < 0.001

Control animals revealed that the majority of the osseous defects were filled with granulation tissue surrounding irregular thin and sparse woven bone trabeculae. However, the other experimental groups demonstrated considerably newly formed bone trabeculae extending from osseous defect margin towards the center surrounding large bone marrow cavities. The bone trabeculae were thicker in the tideglusib hydrogel group than Ethoss group. Numerous osteoblasts observed lining bone trabeculae indicate the active bone formation process (Fig. 8).

##### Six weeks post-surgery

**Fig. 9:**
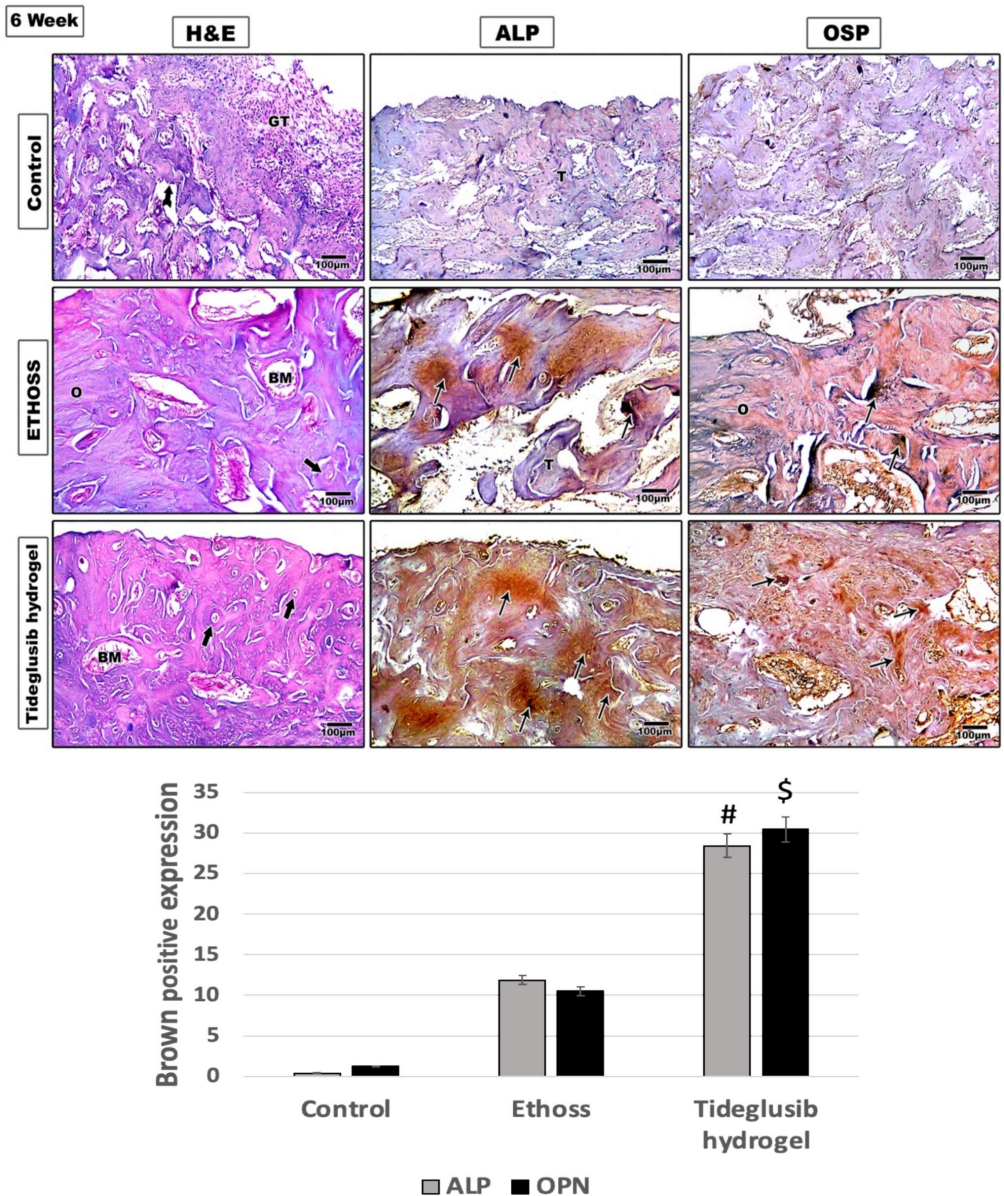
Histologic images of (H&E), Alkaline phosphatase and osteopontin immune staining are shown at 6 weeks after treatment. Granulation tissues still observed in the defect areas in control animals. Ethoss and Tideglusib groups showed bone trabeculae homogenate with the defect borders with relatively narrow marrow spaces. The defect filled with Tideglusib hydrogel shows noticeable formation of osteons (thick black arrow) with extensive brown immune expression in ALP and OPN. T: bone trabeculae; O: old bone; BM: bone marrow; GT: granulation tissue. Arrows indicate ALP and OPN-positive reactions in immune images. Scale bar: 100 μm. The bar chart shows the histomorphometric analysis of ALP and OPN positive expressions at 6 weeks Data are represented as means ± SEM where # and $ means significant in relation to Ethoss and control groups *P* < 0.001

In the control group, the osseous defect exhibited the persistence of a space in the center filled with granulation tissue accompanied by moderate thick immature bone trabeculae. Bone trabeculae were bordered by osteoblastic activity and started to coalesce. The trabecular bone was thicker and denser, in experimental groups when compared to control group. They were also and more mature. However, numerous osteons were seen in tideglusib hydrogel group when compared with Ethoss group, indicating high bone remodeling (Fig. 9).

#### Immunohistochemical staining results

The newly formed bone has been evaluated utilizing the ALP and OPN staining to measure the bone formation and mineralization. After determining the percent of brown positive expression, the following findings were found ALP expression in control group was (0.141 ± 0.002) at 3 weeks & (0.409 ± 0.004) at 6 weeks. In animals treated with Ethoss, ALP expression was (4.92 ± 0.006) at 3 weeks & (11.89 ± 0.008) at 6 weeks while in the tideglusib hydrogel group, ALP expression was (16.66 ± 0.036) at 3 weeks and (28.46 ± 0.002) at 6 weeks. From 3 to 6 weeks, ALP expression significantly increased in the tideglusib hydrogel group than Ethoss or control groups. Moreover, the findings revealed that ALP expression significantly elevated in Ethoss group in comparison to control animals.

Nevertheless, OPN expression in control animals was (0.847 ± 0.003) at three weeks and (1.25 ± 0.21) at six weeks. For the Ethoss group, the expression was at three weeks (5.91 ± 0.005) and at six weeks (10.52 ± 0.003) while in the Tideglusib hydrogel group, OPN expression was (22.33 ± 0.022) at three weeks and (30.48 ± 0.006) at six weeks. OPN expression revealed significant increase in tideglusib hydrogel group compared to Ethoss or control groups after three and six weeks post-surgically. These outcomes imply that the tideglusib hydrogel group might significantly affect bone development and maturation, which would become more noticeable after 6 weeks (Figs. 8 & 9).

## Discussion

Bone formation occur by many different mechanisms which include stimulation of growth factors and regulation of variable biochemical pathways involved in molecular signaling of osteogenesis such as Wnt pathway [45], TGF-β/BMP superfamily [46], notch signaling and hedgehog proteins [47]. The GSK-3 β inhibits bone formation due to suppression of Wnt signaling pathway. Tideglusib irreversibly inhibits the active GSK-3 β protein, and is thought to stabilize β- catenin which is an important downstream target of Wnt signaling [48]. Recent research has focused on improving the osteoconduction and osteoinduction capacity by improving the preparation design or through adding drugs to stimulate growth factors or regulate pathways [49].

Our experiment aimed to assess the effect of local delivery of tideglusib, with its new formulation incorporated with sodium alginate hydrogel on the acceleration of bone regeneration and to compare its effect with Ethoss (alloplastic osteoconductive bone material β-TCP/CS) via in vivo and invitro studies.

Tideglusib is small molecule that irreversibly inhibits GSK-3β, providing sustained activity independent of enzymatic degradation [50], in the present study it has been used in a new hydrogel formulation, as hydrogels have been introduced as promising material due to their excellent properties that can enhance bone regeneration. To our knowledge this formulation of tideglusib have not been tested before as a technique for local delivery of the drug during bone regeneration. Alginate is a natural polysaccharide rapidly transformed into hydrogel via the action of many divalent cations using coupling reactions [51]. Selecting Alginate hydrogel for loading tideglusib was because of its high biocompatibility, low immunogenicity and biodegradability [52].When drugs are loaded into sodium alginate hydrogel, its release is controlled by the scaffold’s degradation and diffusion properties as this hydrogel typically retain structure for 4–6 weeks and fully degrade by 6–8 weeks, depending on its formulation [53]. Consequently, the local activity of tideglusib at the defect site is governed by the controlled release and degradation behavior of the hydrogel matrix.

Ethoss (β-TCP + CS) was used as a comparator as it is widely used in dental/orthopedic applications as a standard bone substitute, it is also a well-documented synthetic graft with predictable resorption and osteoconductive properties, allowing a direct comparison of osteogenic potential without confounding biological variability (e.g., donor-related factors in allografts) [54]. Moreover, studies concluded that β-TCP produced a higher contribution in tibial bone repair than using bone graft substitute alone [55, 56].

Minimal criteria were previously set to define MSCs as they must express CD73, CD90 and CD105 and lack CD45, CD34, CD14, CD11b, CD79a or CD19 surface markers. In our study, BMMSCs were positive for CD90 and CD105, and negative for CD34 and CD14 [57].

In this study, the scanning electron microscope of the hydrogel demonstrated a porous microstructure with interconnected networks which is proposed to enhance cell differentiation and subsequently accelerating bone healing. Zeta potential expressed negative values which possessed positive impact on bony cells’ attachment and proliferation, enhancing the osteogenesis potential and influencing their biological behaviors [58]. Negative zeta potential is an important factor of biocompatible particles, showing higher cell viability [59]. A material with positive zeta potential interacts and/or penetrates cells easily because of their opposite charge as the cells express negative surface zeta potential. So it was believed that the positively charged particles threaten the cell viability [60]. The FTIR spectrum confirms the successful incorporation of both tideglusib and sodium alginate in the hydrogel formulation, as evidenced by the characteristic peaks of both components. The presence of broad O–H bands and strong carboxylate peaks suggests good hydrogel formation with appropriate cross-linking, which is essential for drug delivery applications.

The advancement of hydrogel technology for regenerating maxillofacial bone tissue shows significant promise. Nevertheless, several challenges must be overcome to expand their use in clinical practice. A key challenge involves optimizing cell survival when assessing biocompatibility [61]. Thus, it was necessary to test the viability of BMMSCs incubated with various concentrations of tideglusib hydrogel and Ethoss. After incubation for 72 h the best viability was observed at 20% concentration of the materials, so this concentration was used for subsequent tests in this study.

The ability of cells to react directionally to different chemo attractants, such as chemokines, growth factors, lipids, and nucleotides, was assessed using the migration assay [44]. After 24 h, maximum cell localization was observed for the elute tideglusib hydrogel being significant in comparison to Ethoss treated group. Noteworthy that similar trend of data was obvious after 48 h of migration test. Nair et al. revealed also that tideglusib possess anti-inflammatory activities that improve and hasten the healing response [62].This anti-inflammatory property may give tideglusib hydrogel a superiority in wound healing than Ethoss.

To evaluate the osteogenic differentiation potential of BMMSCs under the impact of the two tested materials, the ARS expression was measured and demonstrated the efficacy of calcium mineralization stage in the osteogenic differentiation of MSCs [63]. The results indicated that tideglusib hydrogel and Ethoss could enhance bone formation with significant difference in comparison to the control group. This result was further confirmed by the in vivo bone defect model which revealed better healing capacity of the tideglusib hydrogel and Ethoss groups more than the control group with superior effect to the tideglusib hydrogel in terms of trabecular thickness and better cavity closure after 6 weeks. ALP, the early osteogenic marker, and OPN expression were also elevated in the tideglusib hydrogel group at 3 and 6 weeks exceeding that of Ethoss.

Ethoss (β-TCP + CS) enhancement of bone formation was in accordance with Flifl et al. who explained the result by increased osteoprotegrin expression in Ethoss treated groups [64]. On the other hand, some researchers revealed conflicting results about β-TCP for bone regeneration which make its usage questionable in many experiment models. For instance, in a mandibular defect model in rats, local application of β-TCP particles was a delay in bone regeneration. This delay was suggested to accelerate fine fibers’ formation within the blood clot altering its structure due to Ca2 + and PO43 − ions release by β-TCP. Therefore, this might interfere with early recruitment of MSCs for bone healing [65].

On the other side enhancement of bone healing was in accordance to Marianne et al. who examined tideglusib's independent effects on femur bone defects, conducting assessments at 7, 14, and 28 days post-surgery. Their findings revealed that tideglusib as a standalone treatment significantly enhanced bone healing compared to controls. They also reported that tideglusib enhanced intramembranous bone repair at the cost of endochondral ossification by inhibiting GSK-3 and activating the cell-based repair mechanism [66].

Enhancing bone regeneration was also reported by Berrin İyilikci, el al. who conducted a research on skull bone defects, testing both xenografts and autografts with and without tideglusib. Results demonstrated that all groups receiving tideglusib with grafts showed substantially improved new bone formation across all measured parameters, indicating that tideglusib works synergistically to amplify graft effectiveness. They explained tideglusib results by the upregulation of BMP 2 and VEGF expression supporting the bone repair mechanism [67].

Research investigating tideglusib's role in bone repair has examined its effects both as a standalone treatment and when combined with different graft materials. In a rat study on calvarial defects treated with tideglusib alongside autografts and gelatin sponges they found that the combination treatment produced significantly better healing outcomes compared to untreated controls, the authors noted that tideglusib enhanced bone mineral density and promoted new bone growth by reducing cell death and stimulating bone-forming cell activity [68].

In a recent in vitro study, osteoblast and macrophage cells were co-cultured in direct contact with or without the presence of tideglusib. ALP activity, histological analyses and the expression of inflammatory-related genes were evaluated. The results revealed that co-culturing with tideglusib increased ALP which was indicative of enhanced osteoblast activity. Tideglusib treatment contributed to the stability of cell morphology within the scaffold, suggesting a supportive environment for cell viability and function, it also significantly reduced the expression of pro-inflammatory markers, such as TNF-α and effectively inhibited osteoclastogenic differentiation [69].

Another recent study utilized CS and tideglusib simultaneously in rabbit proximal tibial defects revealing superior bone healing capacity than using either of them alone [70]. This implies that tideglusib may be a viable substitute for bone graft materials in the treatment of bone deficiencies, either by itself or in combination.

## Conclusion

The results show that adding tideglusib to sodium alginate hydrogel improved the osteogenesis process in rabbit tibial defects. The importance of this is demonstrated in the therapeutic context, especially when using bone replacement materials such as β-TCP. By further exploring tideglusib hydrogel's osteogenic potential, we open the door to novel and potent treatments that can greatly improve the lives of people with bone-related illnesses.

## Data Availability

The corresponding author can be contacted to provide the data of this study.
